# Formative Provider Testing of a New Encounter Decision Aid for Smoking Cessation: Questionnaire Study

**DOI:** 10.2196/32960

**Published:** 2022-04-20

**Authors:** Herul Hollanda De Sa Neto, Ines Habfast-Robertson, Christina Hempel-Bruder, Marie-Anne Durand, Isabelle Jacot-Sadowski, Yasser Khazaal, Ivan Berlin, Kevin Selby

**Affiliations:** 1 Lausanne University Hospital Lausanne Switzerland; 2 Center for Primary Care and Public Health (Unisanté) Lausanne Switzerland; 3 Centre d'Epidémiologie et de Recherche en Santé des Populations University of Toulouse Institut National de la Santé et de la Recherche Médicale Toulouse France; 4 Dartmouth Institute for Health Policy and Clinical Practice University of Dartmouth Lebanon, NH United States; 5 Department of Psychiatry Lausanne University Hospital Lausanne Switzerland; 6 Hôpital Pitié-Salpêtrière Sorbonne University Paris France

**Keywords:** decision aid, smoking cessation, electronic tool, shared decision-making

## Abstract

**Background:**

Smoking cessation is an essential part of preventing and reducing the risk of smoking-associated morbidity and mortality. However, there is often little time to discuss smoking cessation in primary care. Decision aids (DAs) designed for clinic visits (encounter DAs) need to be clear, short, and concise to optimize therapeutic education, increase interaction, and improve the therapeutic alliance. Such a DA for smoking cessation could potentially improve counseling and increase the use of pharmacological treatments.

**Objective:**

We aimed to collect feedback on an electronic encounter DA that facilitates physician-patient interaction and shared decision-making for smoking cessation in primary care.

**Methods:**

We developed an electronic, encounter DA (howtoquit.ch) from a paper version created by our team in 2017 following user-centered design principles. The DA is a 1-page interactive website presenting and comparing medications for tobacco cessation and electronic cigarettes. Each smoking cessation medication has a drop down menu that presents additional information, a video demonstration, and prescribing information for physicians. To test the DA, we submitted a questionnaire to approximately 20 general practitioner residents of an academic general medicine department, 5 general practitioners, and 6 experts in the field of smoking cessation. The questionnaire consisted of 4 multiple-choice and 2 free-text questions assessing the usability or acceptability of the DA, the acquisition of new knowledge for practitioners, the perceived utility in supporting shared decision-making, perceived strengths and weaknesses, and whether the participants would recommend the tool to other clinicians.

**Results:**

In all, 6 residents, 3 general practitioners in private practice, and 2 tobacco cessation experts completed the questionnaire (N=11), with 4 additional experts providing open-text feedback. On the 11 questionnaires, the DA was rated as practical and intuitive (mean 4.6/5), and providers felt it supported shared decision-making (mean 4.4/5), as comparisons were readily possible. Inclusion of explanatory videos was seen as a bonus. Several changes were suggested, like grouping together similar medications and adding a landing page to briefly explain the site. Changes were implemented according to end-user comments.

**Conclusions:**

The overall assessment of the encounter DA by a group of physicians and experts was positive. The ultimate objective is to have the tool deployed and easily accessible for all to use.

## Introduction

Smoking is the leading cause of avoidable, premature death worldwide, with over 8 million deaths per year [1], including 9500 in Switzerland [2]. This excess mortality is explained by increases in cardiovascular and pulmonary diseases and of different types of cancer, including lung cancer (of which 90% is attributable to smoking) [3]. Currently, 30% of men and 21% of women regularly or occasionally smoke in Switzerland, and 18% of the population smokes daily [3]. After many years of decreases, these figures have stagnated over the last decade. Smoking cessation progressively and sustainably reduces the risk of death and the development of smoking-related diseases [4]. Patients wish to discuss smoking during consultations, and more than half of them want counseling about smoking cessation [5].

Primary care physicians play an essential role in promoting tobacco control and smoking cessation. Current international recommendations propose that physicians use every opportunity to discuss smoking during consultations to assess patients’ level of addiction, knowledge, and motivation to stop smoking; to support an eventual quit attempt; and to provide an efficient follow-up [6].

Different pharmacological treatments for smoking cessation are available, including nicotine replacement therapy in the form of bupropion (a dopamine, noradrenaline reuptake inhibitor) and varenicline (a nicotinic cholinergic receptor partial agonist). These treatments, when accompanied by medical follow-up, are associated with quit rates as high as 30% [7] as opposed to rates of 3% to 6% when no aid is provided. Electronic cigarettes (e-cigarettes) are emerging as alternative nicotine delivery devices, are very popular, and seem to be effective aids for smoking cessation [8]. However, long-term data about their safety and efficacy remain limited. Treatments should be prescribed in parallel with intensive follow-up during quit attempts, as repeated consultations have been shown to increase the success of quit attempts [9]. Indeed, the combination of behavioral therapy and pharmacotherapy has demonstrated better results than has either separately [10]. Other nonpharmacological interventions such as hypnotherapy, acupuncture, or mindfulness meditation have less strong evidence of efficacy [11-13].

These different therapeutic alternatives differ by their dosing, mechanisms of action, adverse effects, contraindications, costs, and insurance reimbursement. Giving clear, concise, and appropriate information can be challenging within the time constraints of a consultation. However, guiding patients toward smoking cessation and in the choice of and adherence to a treatment is crucial.

An increasing amount of evidence shows that the relationship between the physician and the patient, often referred to as the therapeutic alliance, plays an important role in treatment adherence and outcomes. Sharing information during shared decision-making can strengthen the physician-patient relationship, involve the patient in the decision-making process, and thus empower the patient to engage in the treatment schedule and adherence. Shared decision-making is described as a 3-step process [14], in which the health care provider formulates the existence of multiple alternatives, describes these options, and finally supports the patient in the assessment of the risks and benefits while exploring their values and preferences. Establishing and strengthening the therapeutic alliance is thereby essential to increasing the number of quit attempts and smoking cessation rates.

Decision aids (DAs) facilitate shared decision-making; they can improve patient knowledge and risk perception while helping to create consistency between the patient’s choices and values [15]. These tools come in different forms: pamphlets, brochures, audiovisual material, web-based apps, or websites. DAs can be employed during consultations (encounter DAs) or can be designed to be used before, after, or independently of clinical encounters [16]. They optimize therapeutic education by increasing interaction [15] and by allowing the patient to be more active in the decisional process [16]. Several DAs have been developed to aid with tobacco cessation, most often as paper or website-based documentation. Their efficacy is supported by a recent systematic review by Moyo et al [17] that analyzed 7 studies evaluating smoking cessation DAs. The systematic review showed a tendency toward an increase in the smoking cessation knowledge, decisional quality, and the number of quit attempts.

A DA was developed in 2017 by our study group [18] through use of an illustrated chart condensing the different methods of delivery, dosing, daily price, efficacy, and principal adverse effects of smoking cessation treatments.

Given the growing evidence about the efficacy of DAs [17] and their impact on the success rate of smoking cessation, this aforementioned tool was reproduced and adapted in an electronic form. In this paper, we aimed to document the characteristics of this new DA and provide results of formative user testing during consultations by general practitioners and experts in the field. The information collected during the testing could lead to changes and improvements to this DA.

## Methods

### Design and Setting of the Study

Herein, we describe the formative testing of a novel DA, exploring the acceptability of the electronic, encounter DA to assess its design, usage, content, and perceived changes in consultation. A formative test is conceived to assess if a product meets users’ needs and to identify potential usability issues [19]. Our assessment was conducted in an urban, academic, primary care practice with approximately 40 residents in Lausanne, Switzerland.

### Ethical Considerations

Ethics approval was not required as no information was collected from patients and the study is considered quality improvement by local definitions [20].

### Description of the DA

The DA used in this study is based on the one developed in 2017 using a user-centered design [21], which is a methodology centered on the user that gathers information about the utilization and progressively adapts the tool in order to optimize usage. Six general practitioners used iterative versions of the model in consultation over several months and helped define which items and criteria seemed to matter most to patients. Price and insurance reimbursement were considered more important than was the possible effect of medications on weight gain. The tool was inspired by Elwyn’s model of shared decision-making [22].

The original paper-based DA was a 1-page table containing essential information to compare available treatments for smoking cessation (Multimedia Appendix 1). Patients and providers identified several inherent limitations of this DA that could not be addressed in its paper format: notably, the small size of the pictures showing each medication, a difficulty in understanding the differences between nicotine replacement therapies, a lack of information about e-cigarettes (vapes), and a lack of prescribing information for physicians.

The new electronic form described in this study contained the same information, including the form of delivery, price per day, main adverse effects, contraindications, and visual analog rating scales that compared the efficacy, addiction potential, and adverse effects of each agent. Comparisons between the treatments were based on data from systematic reviews for efficacy [7] and weight gain [23], on expert opinion for addictiveness, and on published drug information for adverse effects. Finally, short, animated video clips were incorporated, offering animated explanations on the delivery method and tips for use. The new DA also integrated information about e-cigarettes, noting that they are only recommended when medications are not effective and underlining their addictive potential. An image presented examples of vape pens, a box mod (tank system), and a pod mod. Comparisons between e-cigarettes and medications were based on expert opinion.

This DA was designed for use during consultations, making it an encounter DA or a conversation aid. Encounter DAs encourage and directly support patient-clinician conversations when making decisions together [24]. In our opinion, smoking cessation consultations focused on a quit attempt should include an assessment of the patient’s level of addiction that presents the therapeutic alternatives to stop smoking and value clarification. By facilitating the comparison between medications and e-cigarettes, the DA offers implicit value clarification, which can be further explored with the clinician during the consultation. Nonpharmacological treatment alternatives were not included in this DA although the DA should be used in parallel to behavioral counseling during multiple consultations. The combination of behavioral and pharmacological treatments is most effective. We felt that given the limited evidence to support hypnotherapy, acupuncture, and exercise, these should not be included as first-line therapies.

A tool calculating the level of addiction was integrated into the DA, based on the 2 questions from the Heaviness of Smoking Index. Furthermore, a means for patients to specify if they are pregnant or lactating was included. This information is used to create an automatic contraindication alert located above the treatments, written in red, and specifying if the treatment is a contraindication or if it is to be used with precaution. The user has to choose to click on this tool on the left side of the web page to open it.

**Figure 1:**
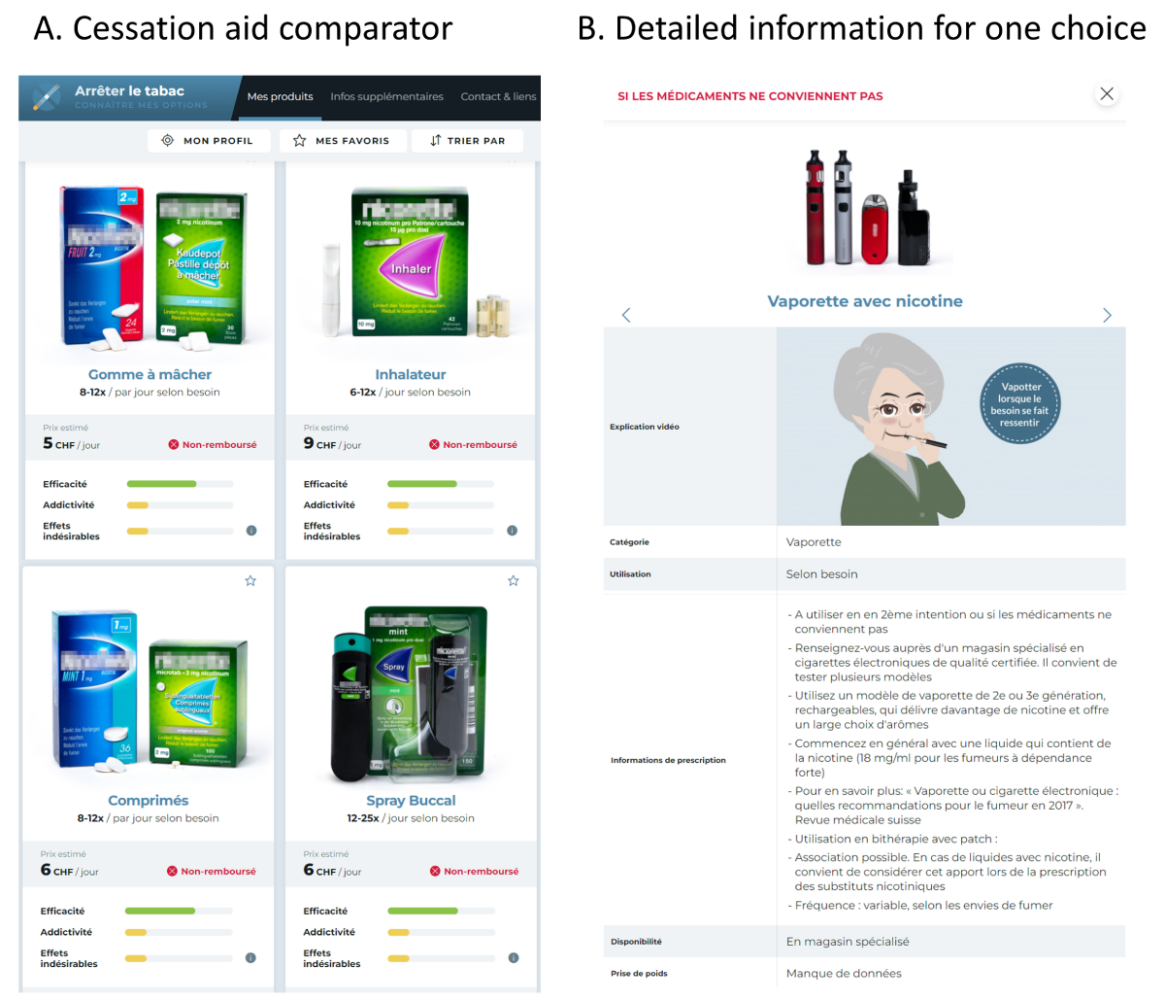
Illustrative screenshots of the decision aid taken on howtoquit.ch. (A) Three of nine available smoking cessation aids in the comparator. (B) Detailed information about one option (electronic cigarettes).

An illustrative image of the decision aid can be seen in Figure 1.

### Population

The population enrolled consisted of potential general practitioner and tobacco cessation specialist end users. A convenience sample of approximately 20 residents from the General Practice of Unisanté (academic service for general medicine) both familiar and unfamiliar with smoking cessation practices were asked to provide feedback after using the DA in consultation at least once with their patients. Participating physicians were free to choose the patients with whom they would use the DA. Physicians used the DA during specialized tobacco consultations or during primary care consultations. Subsequently, 5 selected general practitioners in a private practice were sent an email that explained the study, invited them to familiarize themselves with the DA and, in case of interest, gave them the opportunity to try the tool and give feedback. Finally, the questionnaire was submitted to 6 smoking cessation specialists affiliated with our institution. The expert group included general practitioners, psychologists, and psychiatrists specialized in tobacco cessation or shared decision-making. Two experts in clinical practice were asked to test the DA with their patients and complete the questionnaire.

### Questionnaire

The participants were invited to fill out an online questionnaire. The questionnaire was developed locally and included 4 quantitative and 5 qualitative questions. The quantitative questions assessed the usability or acceptability of the electronic encounter DA, the acquisition of new knowledge for practitioners, the perceived utility in supporting shared decision-making, and whether the participant would recommend the tool to other clinicians. The answers were based on a 5-point Likert-scale (1=strongly disagree, 2=disagree, 3=neutral, 4=agree, 5=strongly agree). We also inquired about the participants’ level of medical training, age, and gender. Two open-text questions asked about the strengths and weaknesses of the DA, and requested recommendations for improvements.

### Analysis

The answers to the quantitative questions based on the 5-point Likert-scale measured the responders level of agreement to the different questions. These results were assembled, and the mean values and SDs were calculated. The open-text questions allowed us to collect information about the enrolled physicians. The questions exploring the weaknesses and strengths of the DA and subsequent recommendations for improvement were considered one by one. They were sorted by categories to allow for better visibility and to help identify recurrences. This brought us to consider the possible changes to the DA.

## Results

We collected the questionnaires described above from 6 residents and 3 general practitioners in private practice who tested the DA. A further 6 smoking cessation experts gave qualitative feedback, 2 of whom also responded to the questionnaire after testing the DA with patients during a consultation. Of the 11 people who completed the questionnaire, 55% (n=6) were women, and all had obtained their medical diploma between 1983 and 2018.

The various groups who provided feedback on the DA and their characteristics are listed in Table 1.

The responders found the electronic interface practical and intuitive, with a mean rating of 4.6 on the 5-point Likert-scale (Table 2). They felt the DA helped improve their knowledge (mean rating 4.4/5). They perceived the tool as facilitating their patients’ decision-making process (mean rating 4.4/5). Finally, globally the physicians strongly agreed that they would recommend the tool to a colleague (mean rating 4.8/5).

The open-text feedback was generally related to content and interface. Content was described as sufficient and relevant. Smoking cessation experts gave advice suggesting to provide more detailed information for e-cigarettes, particularly regarding the fact that e-cigarettes are not considered to be a medication, and that the first-line treatment recommendation include the other presented alternatives only.

Users found the interface to be clear, intuitive, and easy to use. Advice for improvements consisted of making information more noticeable and easier to find. For example, some physicians recommended separating the different types of treatments in groups to facilitate their introduction during the consultation. In the experts’ opinion, the automatic contraindication alert based on the users’ characteristics should be completed with inclusion of other comorbidities and users should be required to answer the questions regarding their level of addiction with a pop-up to draw attention to it. Some physicians recommended separating the different types of treatments in groups to facilitate their introduction. A landing page was suggested as a means of engaging users at their moment of arrival on the website and to provide preliminary information about the tool.

Based on qualitative feedback, the research team ultimately changed the DA in order to improve its content and usability (Table 3).

**Table 1:** Description of user groups providing feedback and their responses.

Group	Participants, n	Type of feedback	Description
General internal medicine residents	6	Quantitative and qualitative	Residents provided feedback after using the DA^a^ in consultation
General practitioners	3 ˙	Quantitative and qualitative	GPs^b^ provided feedback after using the DA in consultation
Smoking cessation experts	6 (2 completed questionnaire, 4 open-text responses)	Qualitative	Local experts provided feedback after exploring the DA

^a^DA: decision aid.

^b^GP: general practitioner.

**Table 2:** Overview of quantitative feedback (N=11).

Questions	1=strongly disagree, n	2=disagree, n	3=neutral, n	4=agree, n	5=strongly agree, n	Mean (SD)
1. Interface is practical and intuitive	0	0	0	4	7	4.6 (0.52)
2. DA^a^ helped me improve my knowledge	0	0	1	5	5	4.4 (0.68)
3. DA enhanced patient's decision-making process	0	0	0	6	5	4.4 (0.52)
4. I would recommend this DA to my colleagues	0	0	0	2	9	4.8 (0.42)

^a^DA: decision aid.

**Table 3:** Changes made to the DA in response to feedback.

Categories of changes to the DA^a^	Users’ comments from open text	Description of changes
Landing page as an entry pop-up	“It was difficult to understand quickly the objective of the website.”	A landing page was added explaining the tool’s objective and advising use with a medical practitioner.
Level of addiction calculator	“Addiction level score isn't noticeable enough. A popup would be better to draw attention on it.”	A pop-up exploring the level of addiction and patients’ specifications (current pregnancy or lactation) was added.
Item layout	“1.It would be simpler to cluster treatments according to their type. 2. It would be better to separate NRT^b^/varenicline/bupropion/e-cigarette^c^.”	Order of the different therapies was adjusted according to a logical setting: short-acting nicotine replacement/long-acting nicotine replacement/combined short and long-acting nicotine replacement/oral medication (bupropion, vernicline)/e-cigarette.
Additions to the content	“E-cigarette: specify that it isn’t a medical treatment and is not recommended as first line treatment.”	Content was upgraded with more detailed information.
Documentation for practitioners	“Would be helpful to better integrate other electronic resources.”	A link was added containing documentation on management of smoking cessation.
Documentation for patients	Should interact with other official websites for smoking cessation in Switzerland	Useful links to official websites giving information, resources, and support for smoking cessation, as well as a local smoking cessation hotline number, were added.
“About us” tab	“It is unclear who made the site and whether they can be trusted”	A tab was added describing the Unisanté tobacco cessation unit, smoking cessation consultation with contact details, and information on project funding and the ongoing randomized trial [25].

^a^DA: decision aid.

^b^NRT: nicotine replacement therapy.

^c^e-cigarette: electronic cigarette.

## Discussion

This paper describes the preliminary user testing and subsequent improvements made to a newly created electronic DA to facilitate decision-making between different types of smoking cessation treatments. Overall, the physician end users found the content to be clear, concise, and useful, and the interface to be practical and intuitive. Based on the self-reported answers to the questionnaire and thus, according to the physicians’ perspective, the tool helped improve users’ knowledge about smoking cessation treatments and assisted patients’ decision-making process.

The development of this new DA was based on user-centered design, an approach originally used to develop products and services [26,27] and used in the health field to create DAs [28]. Hence, a first DA prototype was created based on the content of our first paper-based DA developed in 2017. The user tests allowed us to gather information and comments on this DA. This led to a greater knowledge of users’ needs, as well as the strengths and limitations of the DA. Ultimately, the user feedback identified inherent limitations of the paper format, leading to the elaboration of an electronic format.

The current DA, for use during medical encounters, is not a classic, stand-alone DA with content about disease processes and values clarification. Traditional DA development should follow guidelines from the International Patient Decision Aid Standards (IPDAS) Collaboration, whose extensive criteria allow a standardized assessment of quality in terms of content development and effectiveness [25,29]. Instead, encounter DAs like ours need to be short and intuitive while fitting patient-provider interactions in order to encourage its adoption, as this is dependent on its perceived usability and usefulness by physicians. In this respect, this study chose to specifically focus on the physicians as the DA's end users and testers.

A few existing DAs for smoking cessation have been developed and are mentioned in the literature. Their formats are diverse, but there is a trend toward implementing computerized DAs. Like ours, some of them collect information on patients’ smoking behaviors to provide personalized information and contain written and multimedia-based information [30,31]. One recent study evaluated an app-based DA (iPad app) including, like our DA, information about e-cigarettes, explaining its risks and benefits. This DA was used prior to the clinical encounter. The study showed a higher rate of clinical discussions about smoking cessation, overall patient satisfaction, and acceptability about giving information about e-cigarettes among smokers [32].

Willemsen et al [33] developed a multicomponent DA containing several materials, such as an informative booklet with information on smoking cessation treatments, videos, and samples of some pharmacological treatments. Informative websites exist as well, providing information on smoking cessation treatments and resources functioning as DAs [34]. Most of these DAs are designed to prepare patients for a future clinical encounter. To our knowledge, there are no existing DAs for use during consultations (encounter DAs) that can provide structure, guide information transmission, and promote interactivity and shared decision-making between the practitioner and the patient.

One of the upgrades made to the DA was the inclusion of online material to inform physicians and patients about other existing smoking cessation resources in Switzerland. This includes a reference document with the local epidemiology and recommendations on smoking cessation counseling and treatments (6), web links to the specialized tobacco cessation consultation in Unisanté [35], and 2 official Swiss websites [36] offering information and support to help patients willing to stop smoking. These resources, integrated into the DA, can inform physicians of local resources. It can be easily adapted to other countries and their tobacco-control programs.

After completion of testing and upgrading, the DA has become a new tool for use during a consultation (encounter DA), allowing for better information sharing and ultimately guiding patients into a high-quality decision-making process. Some providers told us of patients’ reactions, but patients were not asked to assess the DA directly.

The strengths of this study include the following: the electronic DA builds on previous experience with a similarly organized paper DA that was tested with patients, responses were collected from target users (clinicians), and feedback was collected at a stage when substantial changes could still be made to the DA. 

There are also several limitations. First, the small sample size might not have provided perspectives from all types of physician users and might have reduced the number of suggestions of other upgrades. However, in the field of usability testing, it is common to have only a few testers as end users, based on the 5-user assumption [37] where a small number of testers can identify the most usability issues although this concept is debated [38]. In our case, the DA content was mostly developed by one of the final end users (medical doctors), tested by physicians during consultations, and reviewed by experts; thus, most of the critical content and usability issues should have been identified. A selection bias is probably present, given that a significant part of the enrolled physicians (general internal medicine residents) worked in our institution. Thus, a more solid assessment would probably have been obtained with a more diversified physician enrollment. Second, we collected the perspectives of general practitioners as end users but not those of patients. Although general practitioners were likely influenced by patient reactions, we are unable to report patient satisfaction with the DA, usability issues from the patients’ perspective, or whether patients felt the DA aided with shared decision-making. However, our DA was designed to be used during a consultation as an encounter DA to help physicians present and describe various treatment alternatives for smoking cessation. In this way, we considered the general practitioners as the end users and the ones most likely to influence uptake of the DA. This study was not designed to evaluate the DA’s effectiveness in terms of number of quit attempts, smoking cessations, or its impact on consultation duration. A clinical trial integrating use of the DA is underway [39]. Finally, we are currently implementing the DA in a study setting where we train general practitioners to use the DA in parallel with intensive counseling; in the future we should clarify for all users that pharmacological treatments and behavioral counseling should be used in parallel.

A new, electronic, encounter DA was generally well received and appreciated by physicians. With their feedback, we were able to implement useful upgrades. This tool will hopefully improve patients’ knowledge and enhance their decision-making regarding smoking cessation. Future objectives include a clinical trial incorporating the DA and making it accessible to all health care providers.

## Appendix

Multimedia Appendix 1 Paper version of encounter decision aid.

## Abbreviations

DA: decision aid
e-cigarette: electronic cigarette
IPDAS: International Patient Decision Aid Standards

## References

[1] Murray C, Aravkin A, Zheng P, Abbafati C, Abbas K, Abbasi-Kangevari M (2020). Global burden of 87 risk factors in 204 countries and territories, 1990-2019: a systematic analysis for the Global Burden of Disease Study 2019. Lancet.

[2] Mattli R, Farcher R, Dettling M, Syleouni M.-E., Wieser S (2019). Die Krankheitslast des Tabakkonsums in der Schweiz: Schätzung für 2015 und Prognose bis 2050. Zürcher Hochschule für angewandte Wissenschaften.

[3] Federal Office of Public Health (FOPH).

[4] Jha P, Ramasundarahettige C, Landsman V, Rostron B, Thun M, Anderson RN, McAfee T, Peto R (2013). 21st-century hazards of smoking and benefits of cessation in the United States. N Engl J Med.

[5] Krebs HK, Radtke T, Hornung R (2010). Raucherberatung in der ärztlichen und zahnmedizinischen Praxis aus Sicht der Rauchenden und ehemals Rauchenden. Tabakmonitoring ? Schweizerische Umfrage zum Tabakkonsum Zürich: Psychologisches Institut der Universität Zürich, Sozialund Gesundheitspsychologie.

[6] (2015). Conseils médicaux aux fumeurs et fumeuses. Vivre sans tabac.

[7] Cahill K, Stevens S, Perera R, Lancaster T (2013). Pharmacological interventions for smoking cessation: an overview and network meta-analysis. Cochrane Database Syst Rev.

[8] Hajek P, Phillips-Waller A, Przulj D, Pesola F, Myers Smith K, Bisal N, Li J, Parrott S, Sasieni P, Dawkins L, Ross L, Goniewicz M, Wu Q, McRobbie HJ (2019). A randomized trial of e-cigarettes versus nicotine-replacement therapy. N Engl J Med.

[9] Lancaster T, Stead L (2017). Individual behavioural counselling for smoking cessation. Cochrane Db Syst Rev.

[10] Stead L, Koilpillai P, Fanshawe T, Lancaster T (2016). Combined pharmacotherapy and behavioural interventions for smoking cessation. Cochrane Db Syst Rev.

[11] Barnes J, McRobbie H, Dong C, Walker N, Hartmann-Boyce J (2019). Hypnotherapy for smoking cessation. Cochrane Db Syst Rev.

[12] White A, Rampes H, Liu J, Stead L, Campbell J (2014). Acupuncture and related interventions for smoking cessation. Cochrane Db Syst Rev.

[13] Maglione MA, Maher AR, Ewing B, Colaiaco B, Newberry S, Kandrack R, Shanman RM, Sorbero ME, Hempel S (2017). Efficacy of mindfulness meditation for smoking cessation: A systematic review and meta-analysis. Addictive Behaviors.

[14] Elwyn G, Frosch D, Thomson R, Joseph-Williams N, Lloyd A, Kinnersley P, Cording E, Tomson D, Dodd C, Rollnick S, Edwards A, Barry M (2012). Shared decision making: a model for clinical practice. J Gen Intern Med.

[15] Stacey D, Legare F, Lewis K, Barry M, Bennett C, Eden K (2017). Decision aids for people facing health treatment or screening decisions. The Cochrane database of systematic reviews.

[16] Montori VM, Kunneman M, Brito JP (2017). Shared decision-making and improving health care. JAMA.

[17] Moyo F, Archibald E, Slyer J (2018). Effectiveness of decision aids for smoking cessation in adults: a quantitative systematic review. JBI Database System Rev Implement Rep.

[18] Jakob J, Cornuz J, Auer R, Jacot SI, Cardinaux R, Selby K (2017). Design and user-testing of a decision aid comparing medications for smoking cessation. Rev Med Suisse.

[19] Russell J, Branaghan E, Hildebrand L (2020). Design for Health: Applications of Human Factors.

[20] Commission cantonale d'ethique sur l'être humain.

[21] Mayo Clinic. Mayo Clinic Shared Decision Making National Resource Center.

[22] Elwyn G, Durand MA, Song J, Aarts J, Barr PJ, Berger Z, Cochran N, Frosch D, Galasiński D, Gulbrandsen P, Han PKJ, Härter M, Kinnersley P, Lloyd A, Mishra M, Perestelo-Perez L, Scholl I, Tomori K, Trevena L, Witteman HO, Van der Weijden T (2017). A three-talk model for shared decision making: multistage consultation process. BMJ.

[23] Farley A, Hajek P, Lycett D, Aveyard P (2012). Interventions for preventing weight gain after smoking cessation. The Cochrane database of systematic reviews.

[24] Kunneman M, Montori VM, Castaneda-Guarderas A, Hess EP (2016). What is shared decision making? (and what it is not). Acad Emerg Med.

[25] The International Patient Decision Aid Standards (IPDAS) Collaboration.

[26] Gould JD, Lewis C (1985). Designing for usability: key principles and what designers think. Communications of the ACM.

[27] Mao J, Vredenburg K, Smith PW, Carey T (2005). The state of user-centered design practice. Commununications of the ACM.

[28] Vaisson G, Provencher T, Dugas M, Trottier M, Chipenda Dansokho S, Colquhoun H, Fagerlin A, Giguere AMC, Hakim H, Haslett L, Hoffman AS, Ivers NM, Julien A, Légaré F, Renaud J, Stacey D, Volk RJ, Witteman HO (2021). User involvement in the design and development of patient decision aids and other personal health tools: a systematic review. Med Decis Making.

[29] Elwyn G, O'Connor Annette, Stacey Dawn, Volk Robert, Edwards Adrian, Coulter Angela, Thomson Richard, Barratt Alexandra, Barry Michael, Bernstein Steven, Butow Phyllis, Clarke Aileen, Entwistle Vikki, Feldman-Stewart Deb, Holmes-Rovner Margaret, Llewellyn-Thomas Hilary, Moumjid Nora, Mulley Al, Ruland Cornelia, Sepucha Karen, Sykes Alan, Whelan Tim, International Patient Decision Aids Standards (IPDAS) Collaboration (2006). Developing a quality criteria framework for patient decision aids: online international Delphi consensus process. BMJ.

[30] Cupertino A Paula, Richter Kimber, Cox Lisa Sanderson, Garrett Susan, Ramirez Rigoberto, Mujica Fernando, Ellerbeck Edward F (2010). Feasibility of a Spanish/English computerized decision aid to facilitate smoking cessation efforts in underserved communities. J Health Care Poor Underserved.

[31] Brunette MF, Ferron JC, McHugo GJ, Davis KE, Devitt TS, Wilkness SM, Drake RE (2011). An electronic decision support system to motivate people with severe mental illnesses to quit smoking. Psychiatr Serv.

[32] Kollath-Cattano C, Thrasher J, Salloum R, Albano A, Jindal M, Durkin M, Strayer Scott M (2021). Evaluation of a smoking cessation patient decision aid that integrates information about e-cigarettes. Nicotine Tob Res.

[33] Willemsen M, Wiebing M, van Emst Andrée, Zeeman G (2006). Helping smokers to decide on the use of efficacious smoking cessation methods: a randomized controlled trial of a decision aid. Addiction.

[34] Brunette MF, Gunn W, Alvarez H, Finn PC, Geiger P, Ferron JC, McHugo GJ (2015). A pre-post pilot study of a brief, web-based intervention to engage disadvantaged smokers into cessation treatment. Addict Sci Clin Pract.

[35] Unisanté. Tobacco Control Unit.

[36] Stop-tabac.ch.

[37] Nielsen J, Landauer T (1993). A mathematical-model of the finding of usability problems. Human Factors in Computing Systems.

[38] Faulkner L (2003). Beyond the five-user assumption: benefits of increased sample sizes in usability testing. Behav Res Methods Instrum Comput.

[39] ClinicalTrials.gov. Combining default choices and a decision aid to improve tobacco cessation (FIRST).

