# Supplementary material for: Formative Provider Testing of a New Encounter Decision Aid for Smoking Cessation: Questionnaire Study
Source: JMIR Form Res. 2022 Apr 20;6(4):e32960. doi: 10.2196/32960 (PMC9069282; doi:10.2196/32960)
Supplement: Multimedia Appendix 1 [file formative_v6i4e32960_app1.pdf]

unisanté
Centre universitaire de médecine générale et santé publique · Lausanne

Arrêter le tabac

CHOIX D'UN AIDE À L'ARRÊT

	MÉDICAMENT	USAGE	PRIX PAR BOÎTE	PRIX PAR JOUR	EFFICACITÉ	ADDICTIVITÉ	EFFETS SECONDAIRES PRINCIPAUX
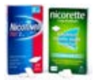	**Gommes à mâcher** Nicotinell / Nicorette ®	**8-12x / jour** selon besoin	~ **20.– CHF** 30 gommes de 2 mg	~ **5.– CHF / jour**	+ +	+	• Irritation bouche et gorge • Hoquet • Nausées
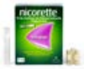	**Inhalateur** Nicorette ®	**6-12x / jour** selon besoin	~ **27.– CHF** 18 cartouches de 10 mg	~ **9.– CHF / jour**	+ +	+	
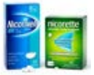	**Comprimés** Nicotinell / Nicorette ®	**8-12x / jour** selon besoin	~ **25.– CHF** 36 comprimés de 2 mg	~ **6.– CHF / jour**	+ +	+	
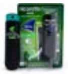	**Spray buccal** Nicorette ®	**12-25x / jour** selon besoin	~ **60.– CHF** 150 pulvérisations	~ **6.– CHF / jour**	+ +	+	
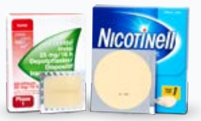	**Patch** Nicotinell / Nicorette ®	**1x / jour** sur 16 ou 24h ou selon besoin	~ **120.– CHF** 14 patches de 15 mg	~ **7.– CHF / jour**	+ +	NON	• Irritation de la peau
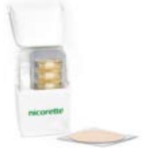	**Combinaison patch & autre substitut nicotinique**	**Patch 1x / jour** + autre substitut selon besoin	~ **120.– CHF** 14 patches et 1 boîte de substitut de courte durée	~ **11.– CHF / jour**	+ + +	+	• Irritation peau, bouche et gorge • Hoquet • Nausées
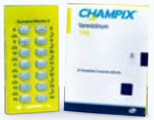	**Varénicline** Champix ®	**2x / jour**	~ **120.– CHF** 56 comprimés de 1 mg ✓ **Peut être pris en charge par l'assurance de base sous certaines conditions**	~ **4.– CHF / jour**	+ + +	NON	• Nausées • Sommeil perturbé • Changement d'humeur
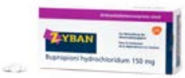	**Bupropion** Zyban ®	**2x / jour**	~ **60.– CHF** 30 comprimés de 150 mg ✓ **Peut être pris en charge par l'assurance de base sous certaines conditions**	~ **4.– CHF / jour**	+ +	NON	• Sommeil perturbé • Bouche sèche • Maux de tête • Changement d'humeur • Troubles digestifs
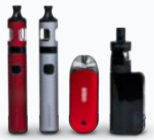	**Cigarette électronique** Vaporette avec nicotine Plusieurs marques disponibles	**Selon besoin**	~ **50.– CHF** Starter kit – diverses marques	~ **4.– CHF / jour**	+ + (+)	+ + +	• Toux • Irritation bouche et gorge • Peut exposer à des composées toxiques à concentrations variables

© unisanté – Version beta 2020 – à ne pas distribuer – **howtoquit.ch**
